# Monocyte populations are involved in the pathogenesis of experimental epidermolysis bullosa acquisita

**DOI:** 10.3389/fimmu.2023.1241461

**Published:** 2023-12-05

**Authors:** Reza Akbarzadeh, Christianna Czyz, Sarah-Yasmin Thomsen, Paul Schilf, Sripriya Murthy, Christian D. Sadik, Peter König

**Affiliations:** ^1^ Department of Rheumatology and Clinical Immunology, University of Lübeck, Lübeck, Germany; ^2^ Institute of Anatomy, University of Lübeck, Lübeck, Germany; ^3^ Department of Dermatology, Allergy, and Venereology, University of Lübeck, Lübeck, Germany

**Keywords:** autoimmune disease, monocyte, CCR2, CX3CR1, intravital imaging

## Abstract

Monocytes play a significant role in the pathogenesis of most inflammatory diseases, including autoimmune diseases. Herein, different subpopulations of monocytes often play differential, partially antagonistic roles, in the regulation of tissue populations. Pemphigoid diseases constitute a group of autoimmune blistering skin diseases featuring a marked infiltration of the dermis with immune cells, including monocytes. The monocyte subsets infiltrating the skin, however, have largely remained elusive. Monocyte adhesion and recruitment into the inflamed tissues are regulated by chemokine receptors, most prominently by CCR2 and CX3CR1. To delineate the involvement of monocyte populations in autoimmune blistering skin diseases, we spatiotemporally monitored the dynamic spectrum of monocyte populations that infiltrate the inflamed skin using multiphoton intravital imaging and reporter mice for chemokine receptors. Experimental epidermolysis bullosa acquisita (EBA) was induced by injection of anti-murine type VII collagen (amCOLVII) IgG into the Csf1r^EGFP^-reporter mice, where circulating myeloid cells, such as monocytes and neutrophils, express an EGFP. EGFP^+^ cells, including neutrophils and monocytes, were present in the skin, immediately after the deposition of the amCOLVII antibody at the dermal-epidermal junction. To investigate the recruitment and involvement of different monocyte-derived cell populations in the disease course further, EBA was induced in CCR2^RFP/+^-reporter and CX3CR1^GFP/+^-reporter mice. A comparable distribution of red fluorescent protein (RFP)^+^ or green fluorescent protein (GFP)^+^ was found in both diseased mice and their respective controls over time, indicating the similar recruitment of monocytes into the skin following the binding of autoantibodies. Experiments were extended to the CCR2^RFP/RFP^-deficient and CX3CR1^GFP/GFP^-deficient mice to determine whether monocyte recruitment and disease severity are compromised in the absence of the receptor. A comparable pattern was seen in the recruitment of monocytes into the skin in both reporter and deficient mice. However, in contrast to similar disease severity between CX3CR1-deficient and reporter mice, CCR2-deficient mice developed significantly less disease than CCR2-reporter mice, as indicated by the percentage of affected area of ears. Collectively, our observations indicate that while CCR2 and CX3CR1 receptors are not involved in the recruitment of monocytes into the skin, CCR2 deficiency is associated with improved disease outcomes in experimental EBA in mice.

## Introduction

Epidermolysis bullosa acquisita (EBA), a rare autoimmune bullous disease, is characterized by the presence of autoantibodies directed against type VII collagen (COLVII) (1). Following the binding of autoantibodies to their antigen, a proinflammatory milieu is generated, in which the infiltration of effector cells is the key pathogenic process initiating and fueling skin inflammation, leading to blister formation (2). In contrast to other cell types, such as neutrophils that are associated with autoantibody-induced tissue damage (2, 3), infiltration kinetics and the contribution of monocytes in skin lesions of EBA in both patients and the experimental models have not been studied in detail.

Bearing wide immune-modulatory and inflammatory potency, monocytes and their infiltration into the inflamed tissue are the hallmarks of several autoimmune diseases, such as systemic sclerosis and rheumatoid arthritis (4–6). Circulating monocytes are heterogeneous, plastic populations that are categorized into at least two subpopulations, classical monocytes (lymphocyte antigen 6 complex locus C (Ly6C)^hi^ in mice, CD14^+^CD16^-^ in humans) and non-classical monocytes (Ly6C^lo^ in mice, CD14^dim^CD16^+^ in humans) with different biological functions (7, 8). The classical, or inflammatory monocytes migrate into inflamed tissue and differentiate further into pro-and anti-inflammatory macrophages that can promote tissue injury or remodeling (9). Conversely, non-classical, or patrolling monocytes scrutinize the blood vessel to maintain vascular homeostasis (10). Chemokines and their receptors play an essential role in the spatiotemporal dynamics of effector cells and their navigation into the peripheral tissues (11). Distinct expression of the chemokine receptors CC-chemokine receptor 2 (CCR2) and C-X3-C motif chemokine receptor 1 (CX3CR1) is associated with monocyte subsets, in which classical monocytes express predominantly CCR2 and the non-classical subsets express lower CCR2 and significantly higher CX3CR1 (12, 13). CCR2 and CX3CR1 have been applied as useful markers to distinguish classical monocytes (CCR2^high^CX3CR1^low^) and non-classical monocytes (CCR2^low^CX3CR1^high^) in both mice and men (14–16).

In the current study, we aimed to resolve the spatiotemporal migration of monocytes in emerging skin lesions. To this end, we used colony-stimulating factor 1 receptor (CSF1r)-reporter mice, where GFP is expressed on the circulating myeloid cells, such as monocytes and granulocytes (17). To uncover further the role of monocyte subsets in EBA disease and the specific function of CCR2 and CX3CR1 in the spatiotemporal dynamics of monocytes, we used CCR2^RFP/+^ (CCR2-reporter) and CX3CR1^GFP/+^ (CX3CR1-reporter) mice to monitor the recruitment of monocyte populations into the inflamed skin tissue. To underscore the specific function of CCR2 or CX3CR1, we generated mice with selective deletion of these chemokine receptors, i.e., CCR2^RFP/RFP^ (CCR2-deficient) and CX3CR1^GFP/GFP^ (CX3CR1-deficient), whose cells express red fluorescent protein (RFP) or green fluorescent protein (GFP) instead of CCR2 and CX3CR1, respectively, and are potentially good candidates to determine the importance of these receptors for the recruitment of monocytes into the tissue (18, 19). We show here that monocyte subtypes infiltrate the inflamed skin during the early stage of the disease in an experimental EBA mouse model, and CCR2 and CX3CR1 chemokine receptors are dispensable for their recruitment. Similarly, monocyte counts are reduced in the blood of human patients with bullous pemphigoid (BP) regardless of chemokine receptors. Although the chemokine receptors are not involved in the recruitment of monocytes into the skin, CCR2 deficiency is involved in protection against experimental EBA, indicating its influence on disease severity.

## Materials and methods

### Mice

B6.Cg-Tg(Csf1r-EGFP)1Hume/J (CSF1r^EGFP+^, CSF1r-reporter) mice with the expression of enhanced green fluorescent protein (EGFP) under the control of the mouse Csf1r promoter, B6.129(Cg)-Ccr2^tm2.1Ifc^/J (CCR2^RFP/RFP^, CCR2-deficient) and B6.129P2(Cg)-Cx3cr1^tm1Litt^/J (CX3CR1^GFP/GFP^, CX3CR1-deficient) mice with a knock-in/knock-out homozygous expression of RFP and GFP replacing the coding sequence of the CCR2 and CX3CR1 loci respectively, as well as wildtype C57BL/6J mice (WT), were obtained from The Jackson Laboratories (Bar Harbor, ME) and imported into the animal facility at the University of Lübeck. CCR2-deficient and CX3CR1-deficient mice were crossed with WT mice to produce CCR2^RFP/+^ (CCR2-reporter) and CX3CR1^GFP/+^ (CX3CR1-reporter) mice respectively, which have the heterozygous expression of RFP and CCR2 or GFP and CX3CR1. Further, these reporter mice were crossed to produce CCR2^RFP/+^×CX3CR1^GFP/+^ double reporter (CCR2×CX3CR1-reporter) mice. Animal protocols were approved by local authorities of the Animal Care and Use Committee of the Federal Ministry of Food and Agriculture of Schleswig-Holstein (Kiel, Germany).

### Human blood samples

This study enrolled 10 consecutive BP patients who were admitted to the Department of Dermatology, University of Lübeck between 2017 and 2019. BP disease was diagnosed based on typical clinical manifestations, detection of linear deposits of IgG and/or complement C3 at the dermal-epidermal junction (DEJ) by direct immunofluorescence of a perilesional skin biopsy, and presence of circulating IgG autoantibodies against BP180 non-collagenous (NC)16A by enzyme-linked immunosorbent assay (ELISA) (20, 21). The study also included a control group with 9 healthy individuals who were age- and sex-matched to the BP patient group. All volunteers gave written informed consent. Approval for this study was obtained from the Institutional Review Board at the University of Lübeck (Lübeck, Germany; ethical proposal numbers 18-046 and 15-051) according to the Declaration of Helsinki. Peripheral blood and biopsies from perilesional skin were collected, and samples with a lack of demographic data or inappropriate processing of initial laboratory specimens which could bias the study were excluded.

### Antibodies

Pathogenic rabbit anti-murine type VII collagen (amCOLVII) IgG was generated as previously described (22, 23). Briefly, white New Zealand rabbits were immunized against the C epitope of COLVII. The IgG was purified, and indirect immunofluorescence analysis was applied to assess its reactivity to murine COLVII on murine skin sections as previously described (24). The amCOLVII antibody was subsequently conjugated with DyLight488 or DyLight594 using commercially available labeling kits (Thermo Scientific, Waltham, MA) according to the manufacturer’s instructions.

Alexa Fluor 647 anti-mouse Ly6G (clone: 1A8), PerCP/Cyanine5.5 anti-human CD14 (clone: 63D3), FITC anti-human CD192 (CCR2) (clone: K036C2), and PE/Cy7 anti-human HLA-DR (clone: L243) antibodies were obtained from BioLegend (San Diego, CA). APC anti-human CD16 (clone: REA423) and PE anti-human CX3CR1 (clone: 2A9-1) antibodies were purchased from Miltenyi Biotech (Bergisch Gladbach, Germany). FITC rat anti-mouse Ly-6C (clone: AL-21) antibody was acquired from BD Bioscience (Heidelberg, Germany). Mouse anti-CD68 antibody was purchased from Thermo Fisher Scientific Inc. (Waltham, MA), and rabbit anti-CD163 antibody was obtained from Abcam Inc. (Cambridge, MA). Alexa Fluor 488 AffiniPure donkey anti-mouse IgG and Alexa Fluor 594 AffiniPure donkey anti-rabbit IgG were received from Jackson ImmunoResearch Laboratories (Cambridgeshire, UK).

### Flow cytometry

Peripheral blood of BP patients or controls was collected, and erythrocytes were lysed with erythrocyte lysis buffer (Buffer EL, Qiagen, Valencia, CA) before counting and staining. Prior to surface staining, 2×10^6^ cells were incubated for 15 min at room temperature with Vioblility 405/452Fixable Dye (Miltenyi Biotech; 130-109-816) diluted 1:100 in phosphate-buffered saline (PBS) to label dead cells. Cells were washed with FC buffer (3% fetal calf serum in 0.01 M PBS, pH 7.2) and blocked with Human TruStain FcX™ (BioLegend, San Diego, CA) and stained for surface markers by incubation with titrated amounts of fluorochrome-conjugated monoclonal antibodies against CD16 APC (1:100), CD14 PerCP/Cyanine5.5 (1:100), HLA-DR PE/Cy7 (1:100), CCR2 FITC (1:40), and CX3CR1 PE (1:40) for 15 min in the dark at 4°C. After incubation, cells were washed two times with PBS before fixing with 4% paraformaldehyde solution (Roti-Histofix, Carl Roth, Germany) for 15 min at room temperature. Subsequently, cells were washed two times using perm wash buffer (BioLegend, San Diego, CA) and resuspended in 500 μL FC buffer before acquisition. All samples were analyzed using a BD FACSAria II flow cytometer (BD Biosciences) and analyzed with FlowJo software (version 10.6.1). The analysis started by gating for single and alive cells. Afterward, gating for CD16 and CD14 allowed the detection of the three different monocyte subsets and gates for the chemokine receptor expression of CCR2 and CX3CR1. To confirm the monocytic cell lineage, HLA-DR positivity was used (**Supplementary Figure 1**).

### Immunohistochemistry and microscopy

For Ly6G/Ly6C staining of mouse skin, cryosections (200 μm thick) were cut from the ear skin of the WT C57BL/6 mice, which were sacrificed 24-, 48-, 72- and 96-hours post-amCOLVII injection. Briefly, hair on the mouse ears was removed by applying hair removal cream (GlaxoSmithKline, Bühl, Germany). Ears were then rinsed with 70% ethanol and amputated with scissors. Using a scalpel, a part of the cartilage was removed by a straight cut along the cranial side of the ear. Ears were then cut medially for better handling and staining. Sections were washed once in PBS to remove any remaining hairs, oriented upright in Tissue-Tek O.C.T. Compound (Sakura Tissue-Tek Xpress, Torrance, CA) in a metal mold, and frozen in liquid nitrogen. Samples were then stained with Alexa Fluor 647 anti-mouse Ly-6G antibody (1:1600) and FITC Rat anti-mouse Ly6C antibody (1:75) overnight at room temperature. After rinsing the sections with Tris-buffered saline (TBS), samples were incubated with a 1:10000 dilution of Hoechst 33342 in TBS for 10 min to stain cell nuclei. Sections were then washed and covered in buffered Mowiol, and mounted with coverslips. Three-dimensional (3D) stacks of stained sections were imaged using a Leica confocal microscope (SP5). FITC Rat anti-mouse Ly6C antibody (emission=490-530 nm) was excited at 488 nm by an Argon laser. Alexa Fluor 647 anti-mouse Ly6G antibody (emission=650-690 nm) was excited at 633 nm using a helium-neon (HeNe) laser. Twenty images of 2 μm thickness were taken for each 3D stack.

To stain the human monocytic cells, biopsies from the perilesional skin of BP patients, and sex- and age-matched healthy donors, were collected. Briefly, 6 μm paraffin sections were deparaffinized, subjected to heat-induced antigen retrieval in the presence of Tris (10 mM)-EDTA (1 mM) buffer (pH 9), washed 3 times with PBS, and blocked with AffiniPure F(ab’)_2_ Fragment donkey-anti-human IgG (Jackson ImmunoResearch Laboratories, Cambridgeshire, UK) in PBS, before blocking with 10% donkey serum (Jackson ImmunoResearch Laboratories, Cambridgeshire, UK). Mouse anti-CD68 (1:600) and rabbit anti-CD163 antibodies (1:800) were applied as primary antibodies. Alexa Fluor 488 donkey anti-mouse IgG (1:500) and Alexa Fluor 594 donkey anti-rabbit IgG (1:500) were used as the corresponding secondary antibodies. Mouse IgG (Thermo Fisher Scientific, Waltham, MA) and unspecific rabbit IgG (Emfret analytics, Würzburg, Germany) were used as corresponding isotype controls for anti-CD68 and anti-CD163 antibodies, respectively. After a washing step, all slides were mounted with 4′,6-diamidino-2-phenylindole (DAPI) Fluoromount-G (Southern Biotech, AL). The sections were visualized and analyzed using the BZ-9000E series microscope (Keyence, Osaka, Japan). Overlay of the signals was conducted by the analysis software BZ-II Analyzer (Keyence, Osaka, Japan). Scoring of positively labeled cells per defined-high power field was performed using Image-J-win64.

### Antibody transfer model of EBA in mice and intravital imaging

The dermis of mice ears was visualized using intravital multi-photon microscopy to examine the binding of amCOLVII IgG at the DEJ, as well as the extent of cell infiltration into the skin. To induce the inflammation and blisters in an antibody transfer-induced EBA, 250 μg of DyLight 488- or DyLight 594-conjugated purified amCOLVII IgG was injected intravenously into the anesthetized mice on day 0. The ears of the mice were scratched lightly with tweezers to promote the extravasation of antibodies at a predefined location. For the long-duration imaging experiments, mice were anesthetized intraperitoneally using 10 µg/kg fentanyl, 16.8 mg/kg midazolam, and 1.7 mg/kg medetomidine hydrochloride. Subsequently, hair removal cream (GlaxoSmithKline, Bühl, Germany) was applied to remove ear hairs. The mouse was placed on a custom-made, heated holder and was kept in a prone position with the head tilted slightly so that the dorsal side of the ear was facing upwards. One ear was fixed lightly with double-sided tape (Tesa SE, Germany), and Vidisic gel (Bausch + Lomb Inc., Laval, CA) was placed in between the ear and a coverslip. The coverslip was fixed lightly with surgical tape (Durapore, Germany) to minimize pressure on the ear. Vital signs of the mouse (O_2_ saturation, heart rate, temperature) were monitored. Images were captured on the outer posterior of the pinna where hair follicles are sparse. Imaging was performed using a TriM Scope II multiphoton microscope (LaVision BioTec GmbH, Bielefeld, Germany) equipped with tunable fs-pulsed lasers and XLPlanN 25×1.05 WMP Objective (Olympus, Hamburg, Germany). Vidisic gel was used as an immersion medium. GFP and DyLigh-488 fluorophores were excited at 920 nm and emissions were detected at 495-560 nm using a photomultiplier tube (PMT). RFP, DyLigh-594 fluorophores, as well as second harmonic generation (SHG) signals, were excited at 1100 nm, and emission signals were collected at >560 nm and 495-560 respectively by separate PMTs. Immediately following the injection of amCOLVII IgG, the visual areas of interest were selected under the microscope in proximity to blood vessels by monitoring of binding amCOLVII IgG to the DEJ and infiltration of the first cells into the skin. Images were acquired from the selected areas during the scan period of each frame with a total of 90 µM Z-stacks at each time point of the experiment for each mouse by starting 20 µM above the DEJ and ending 70 µm below it with a 3 µm thickness between stacks. Image dimensions were 350×350 µm with pixels of 520×520 and the photon frequency was set to 600 Hz. The laser intensity was optimized to reliably image the bright cells and reduce possible laser damage from the excitation of melanin. Image processing was performed using Imaris Software (Bitplane, Zürich, Switzerland). With the use of automatic 3D object tracking and Imaris Spots, the spatiotemporal coordinates of cells were documented. These data were imported into GraphPad Prism 9 for the quantification of cell counts.

Disease severity was scored by determining the percentage of the body surface area affected by skin lesions such as erythema, blisters, erosions, alopecia, and crusts (22). Cell recruitment was monitored on days 0, 1, 2, 4, and 10 in the case of CSF1r-reporter mice and on days 0, 2, 4, 9, and 18 for CCR2-reporter and CX3CR1-reporter, deficient, and double-reporter mice. Control mice were not injected with antibodies but were imaged alongside the diseased mice.

### Statistical analysis

Statistical analysis was performed with GraphPad Prism software (La Jolla, CA). As means of separate groups were analyzed for differences, unpaired two-tailed Student’s t-tests were used. Shapiro–Wilk test was applied to verify the normality of data distribution. Mann-Whitney U test was used to analyze datasets with the non-Gaussian distribution. Dunn’s *post-hoc* test was applied for multiple comparisons between the cell counts of control and diseased mice from all backgrounds. Non-significance was indicated by the symbol “n.s.”. Mean values are indicated on scatter plots. Data are presented as mean ± standard deviation (SD) or as median with interquartile range and *p* < 0.05 was considered statistically significant.

## Results

### Monocytes and neutrophils are present in the early stages of EBA disease onset in mice

**Figure 1:**
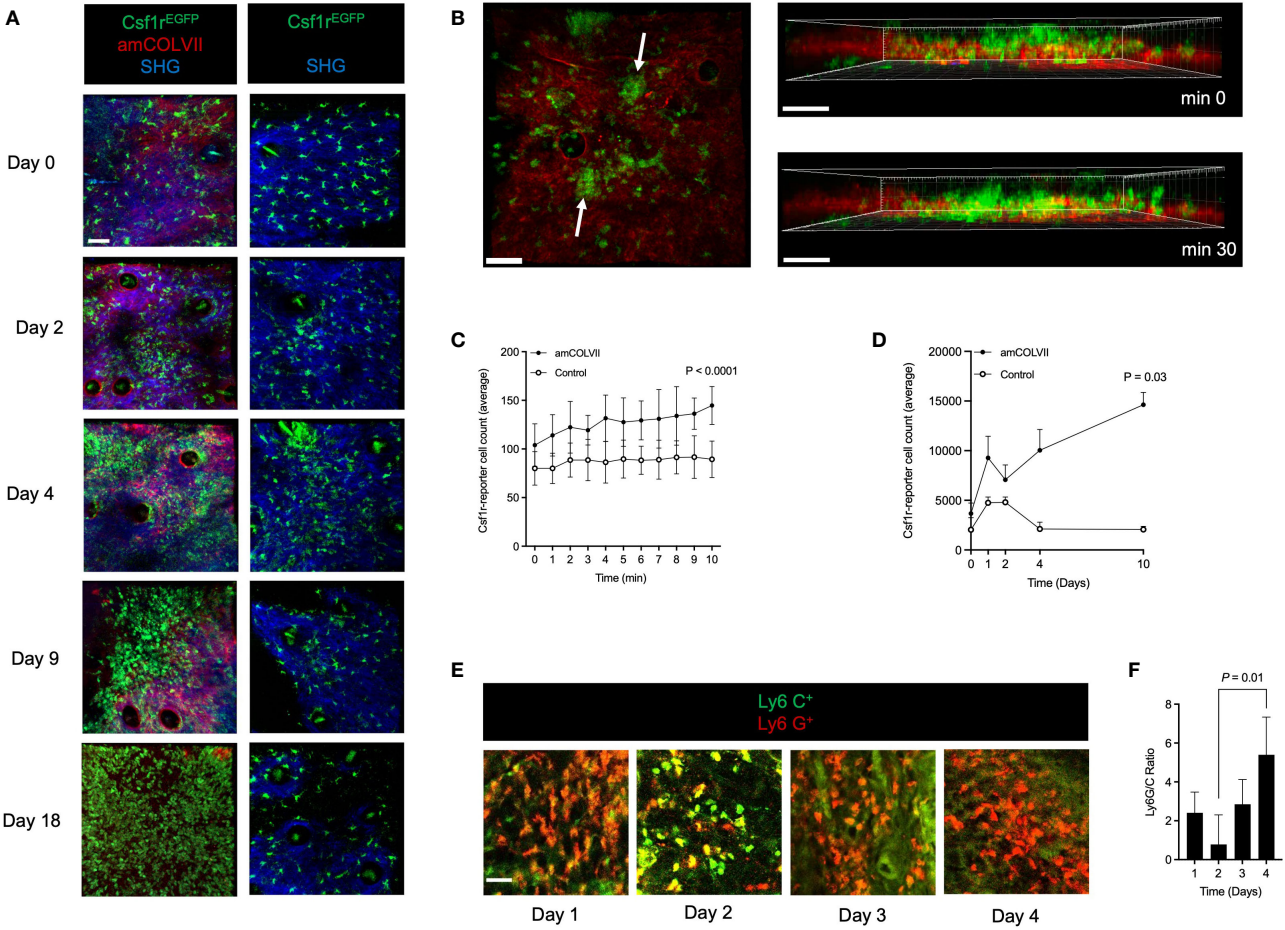
Monocytes and neutrophils are recruited into the skin of mice after amCOLVII antibody injection in an experimental EBA mouse model. **(A)** Representative multi-photon intravital microscopy images of CSF1r^EGFP+^ cells 0, 1-, 2-, 4- and 10-days post-injection with amColVII antibody to the Csf1r-reporter mice (n=8, left) and in control mice (n=3, right). The green color indicates CSF1r^EGFP+^ cells. The red indicates that amCOLVII is only present in diseased (left) mice and not in control (right) mice. SHG is shown in blue. Scale bar: 50 μM. **(B)** Following injection of amCOLVII, CSF1r^EGFP+^ cells infiltrate towards the EDJ in the mouse ear, where the pathogenic antibody is present. White arrows in the left panel indicate the heterogeneous accumulation of the GFP cells. A 3-dimensional image in the right panel shows the spatial orientation and proximity of accumulated antibodies and migrated immune cells in the first 30 minutes. **(C, D)** The average number of CSF1r^EGFP+^ cell count in diseased (amCOLVII) and healthy (control) mice over **(C)** 10 minutes on day 0 and **(D)** 10 days (0, 1-, 2-, 4-, and 10) post antibody injection. **(A–D)** Data presented here are derived from at least 3 monitored areas per mouse. **(E)** Confocal microscopy images of Ly6C^+^(green) and Ly6G^+^ (red) cells stained in ear skin of mice 1-, 2-, 3-, and 4-days post amCOLVII injections. **(F)** The ratio of Ly6G^+^ (Ly6G^high^) cells, which identify neutrophils, to Ly6C^+^ (Ly6G^low^) cells, which identify monocytes, in stained thick sections of C57BL/6 mice 1-, 2-, 3-, and 4-days post amCOLVII injection. **(E, F)** Data are derived from 4 mice per day with at least 3 stained samples. Scale bar: 50 μM. Data are presented as mean ± SD. Statistically significant differences were compared between groups using unpaired t-test analysis. Results of one representative experiment out of three to five independent experiments (ear samples) are shown. amCOLVII, anti-mouse Collagen VII; CSF1R, Colony-stimulating factor 1 receptor; SHG, Second-harmonic generation; SD, standard deviation.

To precisely spatiotemporally resolve inflammatory leukocyte migration in emerging skin lesions, we used CSF1r-reporter mice to chart the dynamics of cell recruitment into the skin of an antibody-transfer EBA mouse model by intravital multiphoton microscopy. To this end, we injected Alexa Fluor 594-labeled amCOLVII antibodies into CSF1r-reporter mice and monitored cell recruitment on 0, 1-, 2-, 4-, and 10 days post-injection. Control mice were not injected with antibodies but were imaged alongside the diseased mice. A gentle mechanical irritation was applied with tweezers on the mice’s ears to amplify the extravasation of pathogenic antibodies (25). This procedure did not visibly affect the epidermis. Shortly after injection, the amCOLVII antibody was observed at the DEJ in mice ears (**Figure 1A**). While few EGFP^+^ cells are present in the skin, immediately after the localization of the amCOLVII antibody at the DEJ, these cells were recruited into the skin in the proximity of antibodies (**Figures 1A, B**). Migrated EGFP^+^ cells form aggregations at several spots in the skin, and the cell counts in these spots increase over time (**Figure 1B**) as judged by the quantification of CSF1r^EGFP+^ cells (**Figure 1C**). Accumulation of these cells was observed over several days, reaching a 7-fold increase on day 10 (**Figure 1D**). Monitoring of the total EGFP^+^ cells in control mice that did not receive pathogenic antibodies indicated a slight increase in the first four days (**Figures 1A, D**). This increase was, however, transient, and only a few scattered populations of cells were registered in the deeper part of the skin, mostly in the dermis.

Given the EGFP expression by both monocytes and neutrophils in CSF1r-reporter mice, recruited cells into the skin could be a mixture of both cells. To discriminate monocytes and neutrophils present in the skin during the early stages of the disease, Ly6G/Ly6C antibody staining was applied in the antibody transfer model of EBA in WT mice. The ear samples were collected from WT mice injected with amCOLVII at 1-, 2-, 3-, and 4 days post-injection. By staining for Ly6G, a marker for neutrophils, and Ly6C, a marker for monocytes, we demonstrated that both cell types are present during initial disease onset (**Figure 1E**). A comparison of the Ly6G^+^ to Ly6C^+^ cells showed that neutrophils are recruited in two waves, on days 1 and 4, whereas monocytes arrive in the greatest numbers on days 2 (**Figures 1E, F**). Together this data suggests that both monocytes and neutrophils are recruited into the skin during the early stages of EBA disease onset in mice.

### CCR2 and CX3CR1 are not involved in the recruitment of classical and nonclassical monocytes in the EBA model

**Figure 2:**
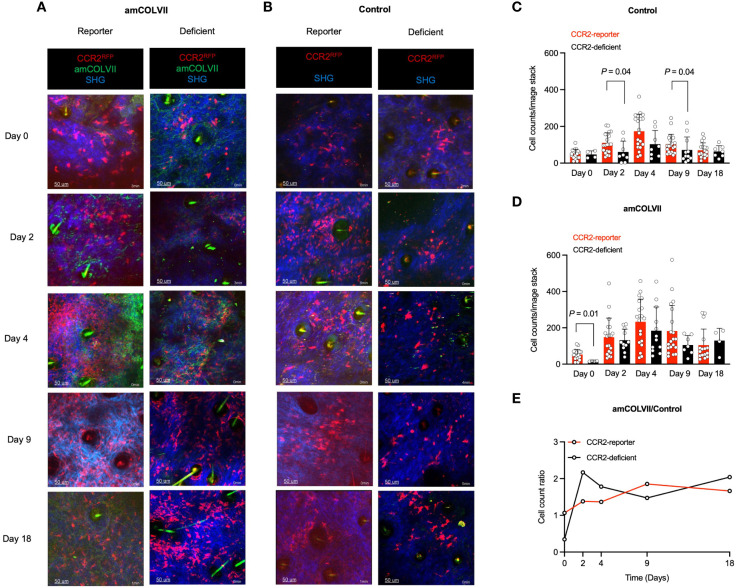
*In vivo* cell recruitment patterns of classical monocytes in mouse ears 0, 2-, 4-, 9- and 18 days post-injection with amCOLVII antibody. **(A, B)** Two-photon microscopy images of RFP^+^ (red) cells at sites of amCOLVII antibody (green) in CCR2-reporter (n=4), CCR2×CX3CR1-reporter (n=6), and CCR2-deficient (n=4) mouse ears injected with in comparison to control (same number of mice). SHG is shown in blue. CCR2^RFP+^ count in the ear skin control **(C)** and diseased **(D)** mice. **(E)** A ratio counts of CCR2^RFP+^ monocytes between diseased and control mice. Data presented here are derived from at least 3 monitored areas per mouse. Scale bar: 50 μM. Data are presented as mean ± SD. Statistically significant differences were compared between groups using the Mann-Whitney test analysis. Results of one representative experiment out of three to five independent experiments (ear samples) are shown. amCOLVII, anti-mouse Collagen VII; CCR2, CC chemokine receptor 2; SHG, Second-harmonic generation; SD, standard deviation.

Having identified monocytes to be present during EBA in mice throughout the disease progression, we sought to investigate the involvement of different monocyte-derived cell populations in the disease course. In mice, monocytes are categorized according to their expression levels of Ly6C and CCR2 as classical (Ly6C^hi^/CCR2^+^) and nonclassical (Ly6C^low^/CCR2^-^) monocytes, with the latter monocyte population typically expressing high levels of the receptor CX3CR1 (26). We investigated the recruitment of classical and nonclassical monocytes into the skin after amCOLVII injection in CCR2, CX3CR1, and CCR2×CX3CR1-reporter mice. Mice were imaged on days 0, 2, 4, 9, and 18 post amCOLVII antibody injection. Non-injected control mice were imaged alongside injected mice to provide information on baseline recruitment due to the mechanical irritation of the skin. As indicated in **Figures 2A, B**, some CCR2^RFP+^ cells were observed in the skin shortly after injection of pathogenic antibody or non-injected control mice (day 0), which was comparable between CCR2-reporter mice in the amCOLVII-injected and control group.

CCR2 is involved in the recruitment of the respective monocyte population into peripheral tissues. To scrutinize the influence of CCR2 molecules in the recruitment of monocytes, CCR2-deficient animals were used, in which the CCR2 locus was completely replaced by RFP expression. In contrast to the healthy mice with the recruitment of few cells until day 4, increased infiltration of CCR2^RFP/+^ cells was seen in mice with or without the CCR2 chemokine receptor (**Figures 2A, B**) with the greatest on day 4 (**Figures 2C, D**). However, in both diseased and control mice, a comparison between CCR2-reporter and CCR2-deficient mice displayed a similar pattern in the recruitment of classical monocytes into the skin (**Figures 2A, B**). Quantification of CCR2^RFP+^ monocytes does not indicate a significant difference between CCR2-reporter and CCR2-deficient mice (**Figures 2C, D**). To detect the effect of disease on the recruitment of classical monocytes, the ratio of cell counts was analyzed. Overall, comparable recruitment of CCR2^RFP+^ monocytes is seen in CCR2-reporter and CCR2-deficient mice (**Figure 2E**).

**Figure 3:**
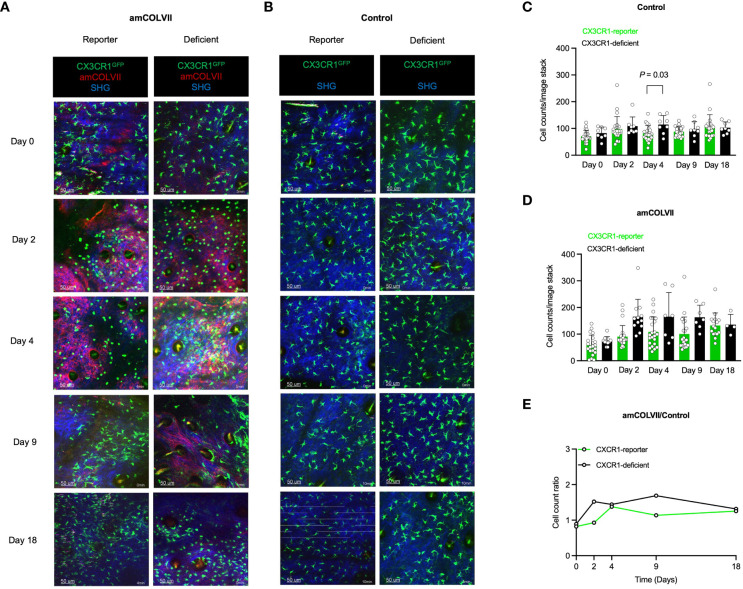
Intravital visualization of nonclassical monocyte recruitment in mouse ears 0, 2-, 4-, 9- and 18 days post-injection with amCOLVII antibody. **(A, B)** Images of CX3CR1-reporter (n=5), CCR2×CX3CR1-reporter (n=6), or CX3CR1-deficient (n=3) monocytes in mice injected with amCOLVII (red) displayed CX3CR1^+^ (green) cells in comparison to the control (same number of mice). SHG is shown in blue. The counts of CX3CR1^GFP+^ monocytes in the ear skin of control **(C)** and diseased **(D)** mice. **(E)** A ratio counts of CX3CR1^GFP+^ monocytes between diseased and control mice. Results of one representative experiment out of three to five independent experiments (ear samples) are shown. Data presented here are derived from at least 3 monitored areas per mouse. Scale bar: 50 μM. Data are presented as mean ± SD. Statistically significant differences were compared between groups using the Mann-Whitney test analysis. Results of one representative experiment out of three to five independent experiments (ear samples) are shown. amCOLVII, anti-mouse Collagen VII; CX3CR1, CX3C chemokine receptor 1; SHG, Second-harmonic generation; SD, standard deviation.

Through interaction with the CX3CR1 receptor, migration of CX3CR1-bearing cells such as monocytes, is facilitated. Hence, a possible effect of this chemokine receptor was considered in the recruitment of the CX3CR1^+^ monocyte population. To identify non-classical monocytes, CX3CR1-reporter mice were used and the involvement of CX3CR1 in the recruitment of CX3CR1^GFP+^ monocytes was analyzed. In contrast to classical monocytes, visualization of non-classical monocytes exhibited no difference in the recruitment of these cells between CX3CR1-reporter and CX3CR1-deficient mice neither in diseased mice nor in controls (**Figures 3A, B**). This was further confirmed by no significant differences in cell counts between control and injected mice (**Figures 3C, D**). Confirming these data, the ratio of CX3CR1-reporter and CX3CR1-deficient remains similar between diseased mice and controls (**Figure 3E**). Taken together, these data indicate that deficiency in chemokine receptors CCR2 and CX3CR1 does not play a role in the recruitment of classical and non-classical monocytes into the inflamed skin of an experimental model of EBA.

### CCR2 deficiency, but not CX3CR1, contributes to partial protection against experimental EBA in mice

**Figure 4:**
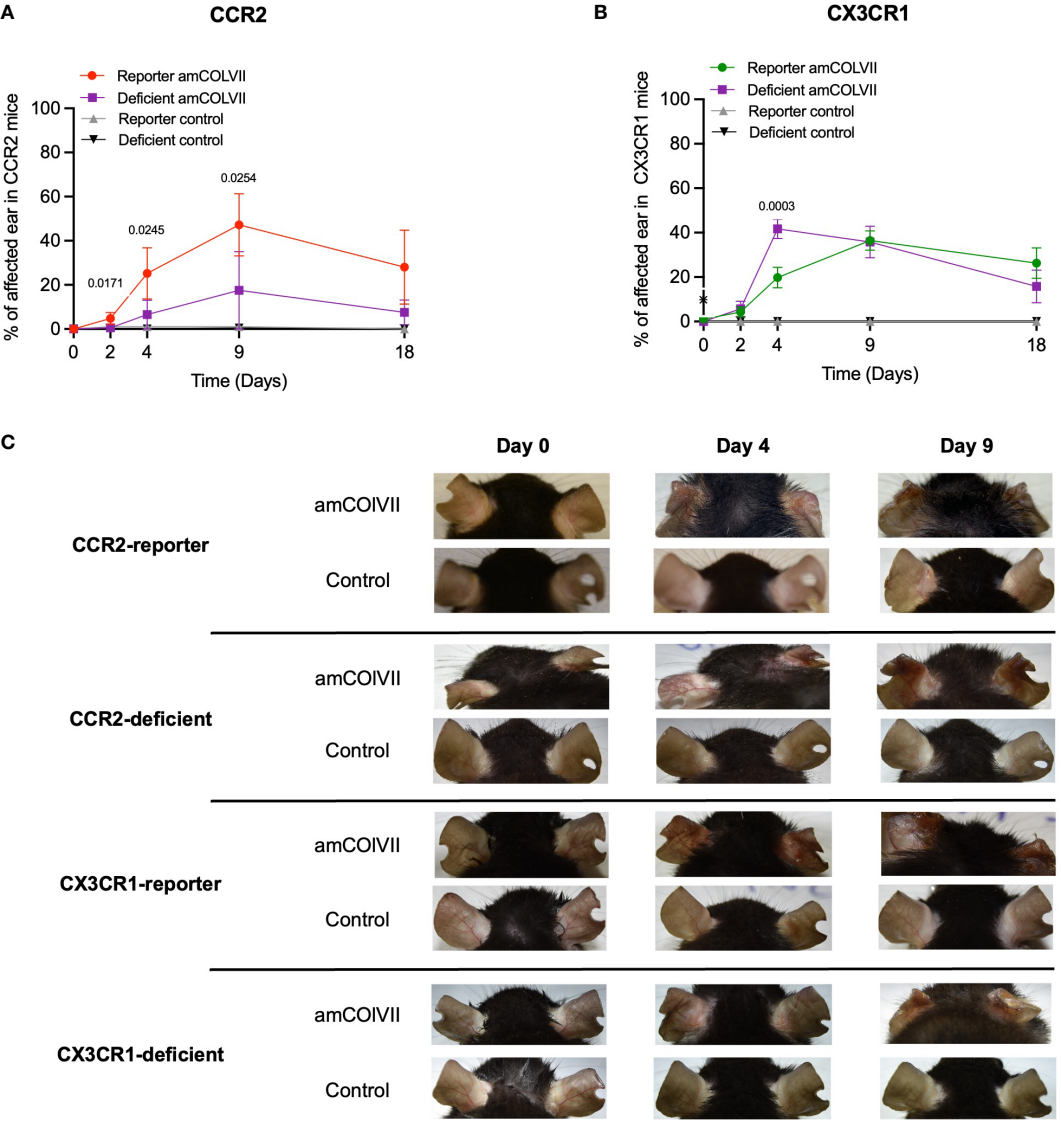
Disease phenotypes in CCR2 and CX3CR1 reporter and their respective deficient mice in an EBA mouse model after amCOLVII antibody injection. CCR2-reporter (n=4), CX3CR1-reporter (n=5), CCR2×CX3CR1-reporter (n=6), CCR2-deficient (n=4), CX3CR1-deficient (n=3) mice were injected with an amCOLVII antibody, and disease severity of the ears was assessed on 0, 2-, 4-, 9- and 10 days post-injection. Control mice were not injected. Disease phenotype development is shown as a percentage of ear skin surface area affected by blistering (crusting, erosions, and/or abrasions). **(A)** The time course of clinical disease development in CCR2-reporter and CCR2-deficient diseased and control mice. Data are presented as mean ± SD. Statistically significant differences were compared between groups using unpaired t-test analysis. **(B)** The time course of disease phenotype development in CX3CR1-reporter and CX3CR1-deficient diseased and control mice. **(C)** Ears of the CCR2-reporter and CCR2-deficient mice.

Classical CCR2^+^ and non-classical CX3CR1^+^ cells have been previously identified as inflammatory and resident monocytes, respectively, which play a role in inflammation and homeostatic functions (14). Therefore, we hypothesized that the recruited inflammatory CCR2^+^ monocytes could be involved in the pathogenesis of EBA disease and CCR2-deficient mice should show a less severe skin disease as compared to the WT. Since CCR2 and CX3CR1 are not functional in CCR2-deficient and CX3CR1-deficient mice, the influence of these chemokine receptors on the clinical phenotype was investigated in experimental EBA, where the deficient mice were compared with its corresponding reporter control. Both reporter and deficient mice started to develop clinical symptoms such as erosions, alopecia, and crusts on day 2 after the injection of the pathogenic amCOLVII antibodies (**Figures 4A, B**). Increased severity of disease based on the total area of affected ears was observed over time, with the strongest effect seen on day 9 in the EBA model (**Figures 4A, B**). Control mice without injection of pathogenic amCOLVII IgG did not develop the disease. While a significant difference was observed between CCR2-reporter and CCR2-deficient mice, disease severity remained similar between CX3CR1-deficient and reporter mice (**Figures 4A–C**). These data indicate that although the chemokine receptor CCR2 is dispensable in the recruitment of monocytes into the skin, it still plays a role in disease severity.

### Circulating monocyte proportion regardless of chemokine receptors CCR2 and CX3CR1 is altered in BP in human

**Figure 5:**
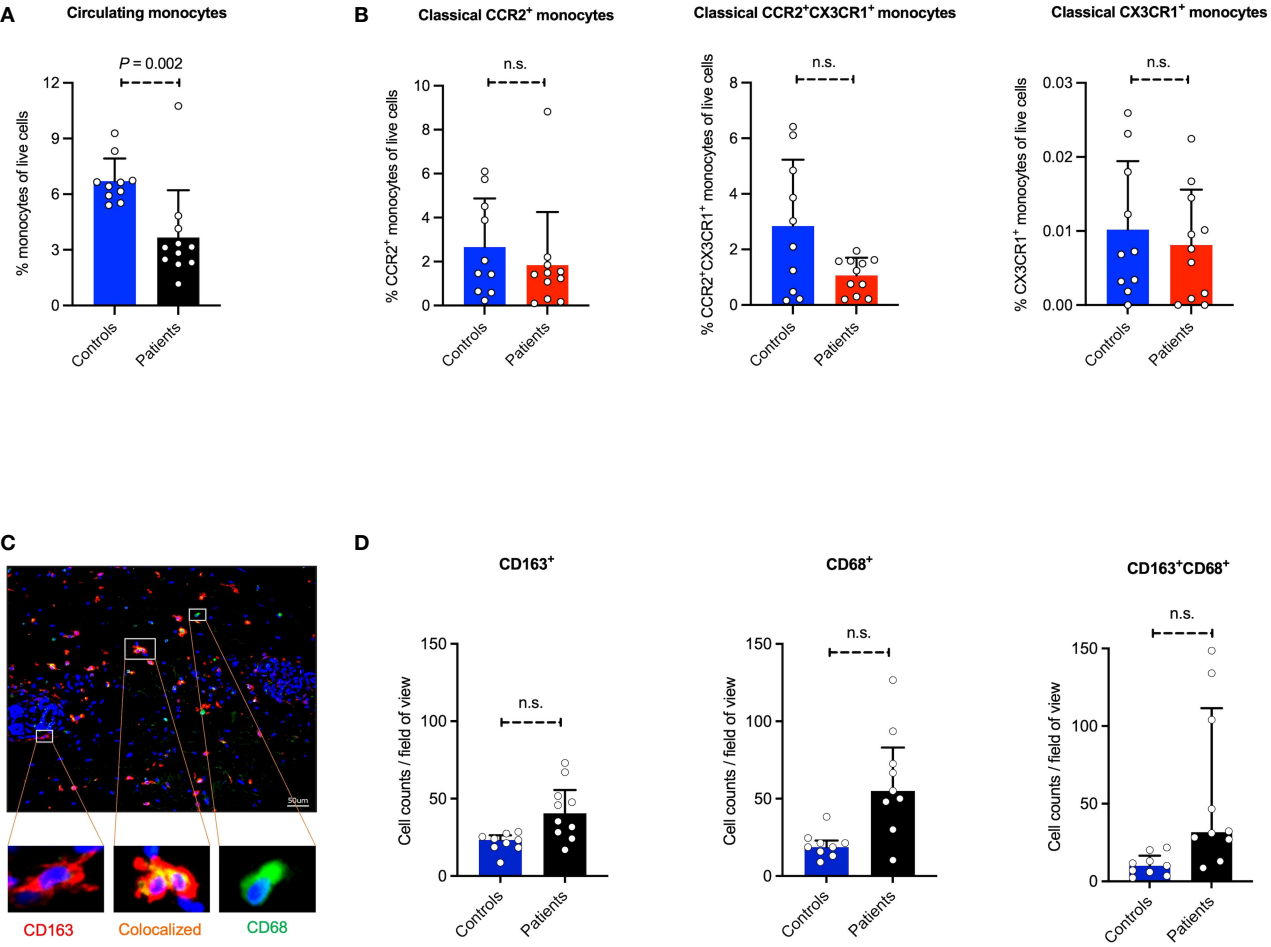
Prevalence of monocyte/monocyte-derived population in human blood and skin. Absolute cell counts of monocyte populations and subpopulations were determined in peripheral blood in 10 BP patients and 9 healthy controls. **(A, B)** The monocytes and monocyte subpopulations (classical CCR2^+^, classical CCR2^+^CX3CR1^+^, and classical CX3CR1^+^ monocytes) in human peripheral blood samples using flow cytometry measurement. **(C, D)** The human skin section performed immunostaining of CD68, CD163, and CD68/CD163. Skin biopsies were taken from the lesions and healthy skin, stained, and microscopically counted. The CD68 and CD163 single-positive, as well as double-positive cells, are indicated. Data are presented as mean ± SD. Statistically significant differences were compared between groups using the Mann-Whitney test analysis. Scale bar: 50 µm.

Since we observed the recruitment of classical monocytes in a mouse model of bullous pemphigoid (BP)-like EBA disease, we next investigated if monocytes are involved in human BP. Patients with EBA are extremely rare. The clinical, histologic, and immunohistologic features as well as immune profiles, binding pattern of autoantibodies, and mechanisms of cell recruitment in BP disease are like what is seen in EBA. We therefore analyzed the number of blood monocytes between the BP patients and healthy individuals. As indicated in **Figure 5A**, our data show that the percentages of monocytes are significantly lower in the blood of the patients compared to the control subjects. Further analysis of classical monocyte subpopulations indicated no difference in the percentages of CCR2^+^, CCR2^+^CX3CR1^+^, and CX3CR1^+^ monocytes between BP patients and healthy controls (**Figure 5B**). This data suggests that the egress of monocytes from the blood is not regulated by the expression of these chemokine receptors.

### Monocytes and macrophages are infiltrating the inflamed skin of human BP patients

Since a reduced number of classical monocytes in the systemic blood circulation of BP patients was observed, we hypothesized that these monocytes are being recruited into the inflamed skin. Herein, monocytes/macrophages (MOMA) staining was applied on the human skin biopsies, where CD68, CD163, and double staining of CD68/CD163 were performed (**Figures 5C, D**). Of note, the number of activated MOMA, which are represented by the CD68-CD163 double-positive stained cells, is significantly increased in perilesional skin sections of BP patients compared to the control group. Compared to the healthy controls, the expression of CD163, a mononuclear phagocyte-restricted phagocytic receptor expressed on activated monocytes and macrophages, as well as CD68, a pan-macrophage marker, in diseased skin is consistent with the recruitment of monocytes into the inflamed tissue.

## Discussion

The recruitment of effector cells into the injured tissue is the key pathogenic process initiating and fueling skin inflammation in many autoimmune diseases such as rheumatoid arthritis, systemic sclerosis, and pemphigoid diseases. In pemphigoid diseases, autoantibodies are considered the direct cause of disease pathology. Although the key requirements for antibody-mediated diseases have so far been identified, the spatiotemporal dynamics of effector cell recruitment from the blood vessel lumen to the skin as well as the molecular cues choreographing this recruitment, are poorly understood. We investigated here whether these chemokine receptors are essential for monocyte recruitment into the skin, and contribute to the development of the disease manifestation in an experimental mouse model of EBA. Our results indicate a dispensable role of CCR2 or CX3CR1 chemokine receptors for monocyte recruitment to the site of antibody-binding and inflammation in mice. Our data derived from human BP samples also confirmed monocyte recruitment into the patient’s skin compared to healthy individuals. However, deficiency in CCR2 molecules ameliorates the development of experimental EBA in mice.

Establishing tissue inflammation requires the directed migration of effector cells from the vessel lumen to the emerging inflammatory focus and the congregation of cells at this site. Monocyte recruitment is observed following various forms of tissue injury, which represents a characteristic hallmark of inflammation. They have been implicated in the pathogenesis of several autoimmune diseases, executing diverse, partially opposing functions in both the emergence and the resolution of tissue inflammation (5, 6). Within the context of pemphigoid diseases such as EBA, the role of monocytes has hardly been addressed. It has been shown that monocytes are the dominant cell type in early skin lesions of human BP (27), and CD163^+^CD206^+^ M2 macrophages and FcϵRI^+^CD207^+^ Langerhans cells infiltrate lesional BP in significant numbers (28, 29). The C-X-C motif chemokine ligand 10 (CXCL10) has been suggested to drive BP by inducing the release of matrix metalloproteinase-9 (MMP-9) from monocytes and neutrophils (30). Recently, Graauw et al. confirmed that monocytes can increase neutrophil-induced blister formation in an ex vivo model of bullous pemphigoid (31). Albeit the presence of monocytes in the diseased tissues has so far been confirmed, these data are mostly based on immunostaining or *in vitro* and ex vivo analysis and do not provide precise information about the dynamics of monocyte populations in recruitment towards the injury. Using multi-photon microscopy, we charted infiltration of myeloid-derived cells such as monocytes and neutrophils in CSF1r-reporter mice. Since we have previously visualized the immediate and inhomogeneous distribution of amCOLVII autoantibodies along the DEJ (25), a local stimulation was performed on the mice ears to induce local antibody extravasation and restrict antibody binding to a defined skin area to facilitate visualization. Our imaging data indicate early recruitment of CSF1r^EGFP+^ cells, predominantly neutrophils and monocytes, followed by a second wave of cell recruitment. Complemented with Ly6C/Ly6G staining for uncovering the identity of recruited cells, we show that both neutrophils and monocytes are rapidly infiltrated into the skin, where neutrophils and monocytes accumulate in the skin in their greatest number at 48 and 72 hours, respectively. In agreement with these data, we recently confirmed rapid dermal recruitment of neutrophils in an experimental EBA model in LysM^EGFP^ mice (25), that express EGFP under the control of the endogenous lysozyme M promoter (32). These data suggest that both monocytes and neutrophils are recruited into the skin during the early stages of EBA disease onset in mice. The observation of two waves of cell recruitment agrees with the model of initial recruitment of limited numbers of cells possibly due to mechanical irritation that we used to induce localized antibody extravasation. These initially recruited cells then could bind to autoantibody at the DEJ which triggered the release of inflammatory mediators that led to the recruitment of larger numbers of cells (33). In accordance with this model, we have observed a rapid recruitment of lower numbers of GFP^+^ cells in control mice that did not receive autoantibody.

The directed migration of effector cells is a complex process consisting of several distinct steps, including trans-endothelial migration (TEM), interstitial tissue migration, and, finally, arrest before effector functions can be executed (34). Evidence has accumulated that effector cell migration is often choreographed by temporally and spatially organized networks of tissue environment- and pathogenic stimulus-dependent molecular guidance signals including chemokine-chemokine receptor interactions and cell adhesion molecules (34–38). Herein, CCR2 is believed to be involved in the migratory functions of monocyte subsets. It has been shown that following the binding of monocyte chemoattractant protein-1 (MCP-1) to its receptor CCR2, the MCP-1/CCR2 signaling pathway contributes to the recruitment of monocytes to the site of inflammation (39, 40). In line with these findings, other investigations have shown that CCR2-deficient monocytes do not migrate in response to MCP-1 and have less capability of adhering to the endothelium, indicating the involvement of CCR2 in firm adhesion of monocytes to the endothelium for exiting the blood vessels (41). In contrast to this data, in the present study, a comparable pattern was observed in the recruitment of CCR2-reporter and CCR2-deficient monocytes into the skin in the experimental model of EBA. These results can be interpreted by the different effects of CCR2 in monocyte release from bone marrow to the blood or migration of monocytes from blood into the tissue. Despite the existence of evidence that CCR2-signaling is important for monocyte egress from the bone marrow into the bloodstream (42), its contribution to the infiltration of monocytes into the tissue remains controversial. Challenging the traditional concept of CCR2 function, some observations indicate that a reduced number of CCR2-deficient monocytes in the inflamed tissue may not solely be due to the essential role of CCR2 in the recruitment of monocytes into the tissue, as there is a remarkable decrease in the ratio of CCR2-deficient to WT monocytes in circulation (43). Brühl et al. also indicated increased recruitment of monocytes into the joints after CCR2 blockade, suggesting that monocyte infiltration can be independent of CCR2 (44). Hence, our findings agree with previous data suggesting that CCR2 only indirectly influences monocyte infiltration into the infected tissues (45, 46).

To translate the effect of CCR2 in the migration of human monocytes in pemphigoid diseases, we further monitored the monocyte subsets in the blood and perilesional skin of BP patients. Our results indicated that although the absolute count of monocytes drops in patients compared with healthy individuals, the number of subpopulations in classical monocytes remains similar between BP patients and healthy controls. A comparable number of CCR2^+^ classical monocytes compared to other monocyte subpopulations indicates that no selective monocyte draining from blood occurs based on chemokine receptors, suggesting that CCR2 is dispensable for the recruitment of monocytes to the skin. However, although the chemokine receptors CCR2 and CX3CR1 do not contribute to the recruitment of monocytes into the skin in our mouse model, we could show that CCR2-deficient mice develop less disease than reporter mice, suggesting a role for CCR2 in disease severity. This could be due to the plastic nature of monocytes that are crossly differentiated into the different populations upon infiltration into the tissue. Circulating inflammatory monocytes infiltrate into inflamed tissue and are polarized into phagocytic pro-inflammatory macrophages at the early disease stages, which later switch their phenotype to an anti-inflammatory subset that helps the resolution of inflammation (10, 47, 48). CCR2 deficiency can alter the balance between inflammatory and anti-inflammatory macrophages, in which CCR2 deficiency blocks the excessive converting of macrophages to a proinflammatory phenotype (49). Hence, this is not beyond the expectation that CCR2-deficient monocytes might migrate into the skin and restore the macrophage polarization balance to favor anti-inflammatory cells, reducing the disease severity. Another possibility for disease attenuation could be the involvement of CCR2 signaling in cells other than monocytes and macrophages that play a role in disease development. For instance, in an animal model of allergic contact dermatitis, it has been shown that CCR2/CC-chemokine ligand 2 (CCL2) signaling is activated in skin neurons that promote itch behavior (50). Since mechanical irritation such as scratching and itching implicates the initiation phase of disease development in various autoimmune skin diseases such as EBA (51), CCR2 deficiency might decrease the scratching events and thereby ameliorate clinical disease manifestation. However, defining the exact role of CCR2 in EBA and BP requires further investigation.

We observed a significant increase in the number of activated monocytes/macrophages (MOMA), represented by the CD68-CD163 double-positive stained cells in perilesional skin. This increase in MOMA in the skin lesions of BP patients is compatible with the declining monocytes in the systemic circulation, where monocytes most likely leave the blood vessels to infiltrate the inflamed skin. Although MOMA markers do not show the exact composition of the specific monocyte/macrophage populations, it is reasonable to assume that the blood monocytes could have migrated into the skin and differentiated to activated macrophages or even function as effector cells. For example, it has been shown that CD163^+^ activated tissue macrophages are increased in several autoimmune diseases such as BP, pemphigus vulgaris, and systemic sclerosis (52, 53). CD163 expression can be increased both by factors inducing the differentiation of monocytes to macrophages and/or by anti-inflammatory mediators such as interleukin (IL)-10 or glucocorticoids (54).

Our study is not without limitations. We could not resolve the spatiotemporal relationship between neutrophils and monocytes at the site of antibody binding. Using dual reporter mice with labeled neutrophils and classical or non-classical monocytes could be helpful in simultaneously charting the kinetics of cell migration in emerging skin lesions. The second limitation was the lack of samples from patients with the inflammatory form of EBA, as this is an extremely rare disease. However, the mouse model we used is very similar to BP with respect to immunologic mechanisms. Therefore, we anticipate that the results we obtained by analysis of samples of BP patients are comparable to results we would have obtained from patients with the inflammatory form of EBA. Despite these limitations, our findings demonstrate a substantial recruitment of monocytes in the skin following the binding of autoantibody to the DEJ. Although the role of monocytes in blistering diseases remains to be determined, they could serve as potential pharmacological targets. The observation that CCR2, despite being dispensable for monocyte recruitment, modulates disease severity, might help in developing potential therapeutic strategies targeting this receptor pathway in EBA and BP.

## Data availability statement

The original contributions presented in the study are included in the article/**Supplementary Material**. Further inquiries can be directed to the corresponding author.

## Ethics statement

Approval for this study was obtained from the Institutional Review Board at the University of Lübeck (Lübeck, Germany; ethical proposal numbers 18-046 and 15-051). The studies were conducted in accordance with the local legislation and institutional requirements. The participants provided their written informed consent to participate in this study. Animal protocols were approved by local authorities of the Animal Care and Use Committee of the Federal Ministry of Food and Agriculture of Schleswig-Holstein (Kiel, Germany). The study was conducted in accordance with the local legislation and institutional requirements.

## Author contributions

RA, CC, and S-YT participated in performing the experiments. RA, CC, S-YT, PS, and SM analyzed the data. RA, PK, and CS contributed to the conception and design of the study. RA and CC wrote the first draft of the manuscript. RA provided the final version of the manuscript, and PK and CS revised it. All authors contributed to the manuscript revision, read it, and approved the submitted version.

## Supplementary material

Supplementary Figure Flow cytometry gating strategy for monocyte subpopulations in human peripheral blood samples. Cell debris was excluded during measurement. Cells were separated from counting beads and gated in FSC/SSC, single cells gated in SSC-A/SSC-H. Cells were plotted for their CD14 and CD16 expression and three distinct subpopulations of monocytes, i.e., classical, non-classical, and intermediate were analyzed. Resulting populations were subsequently plotted for CCR2 and CX3CR1 expressions. And finally, cells were selected for HLA expression.
