# Supplementary figures and images for: Monocyte populations are involved in the pathogenesis of experimental epidermolysis bullosa acquisita
Source: Front Immunol. 2023 Dec 5;14:1241461. doi: 10.3389/fimmu.2023.1241461 (PMC10728641; doi:10.3389/fimmu.2023.1241461)

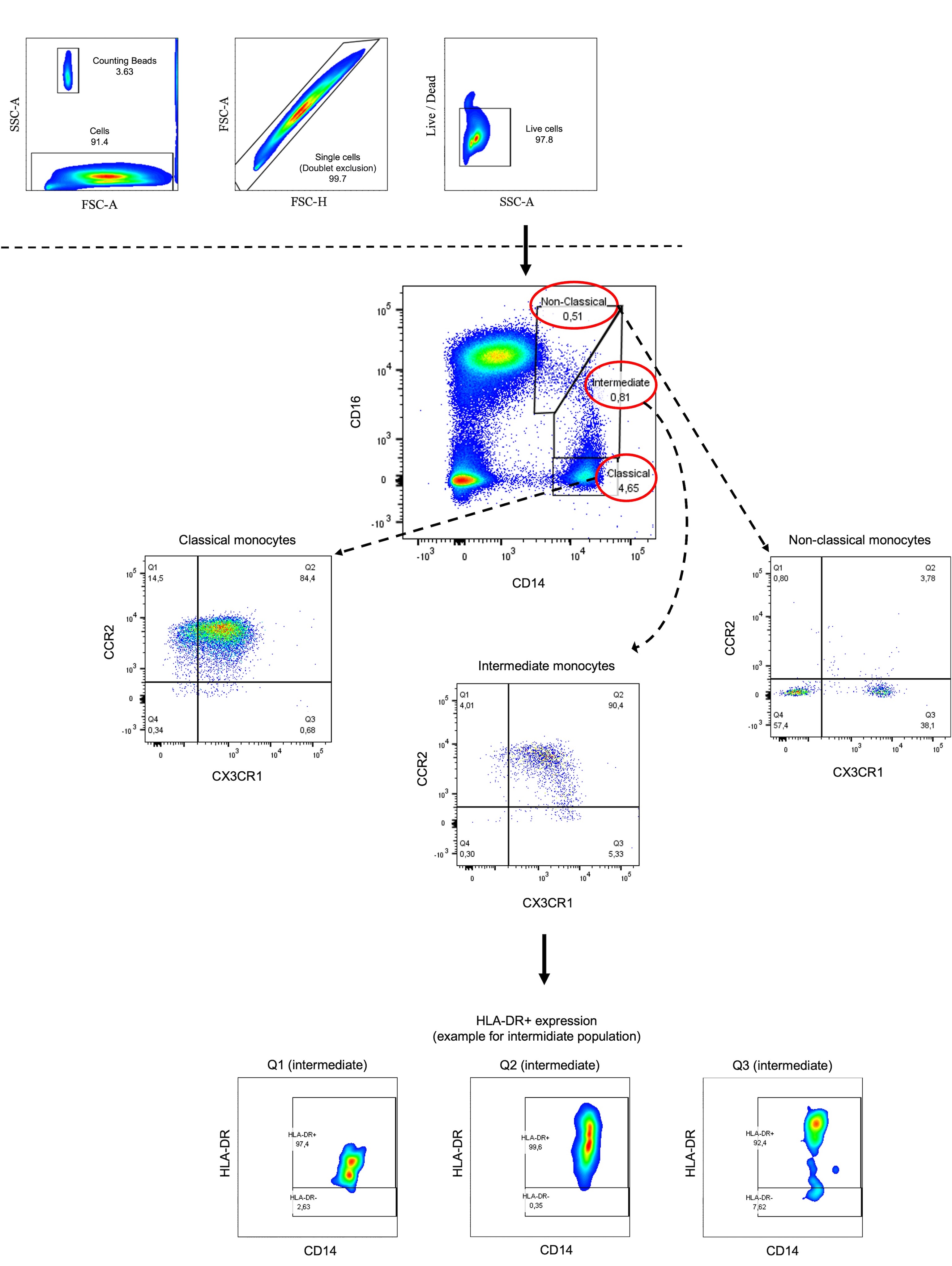

Supplement: Supplementary Figure — Flow cytometry gating strategy for monocyte subpopulations in human peripheral blood samples. Cell debris was excluded during measurement. Cells were separated from counting beads and gated in FSC/SSC, single cells gated in SSC-A/SSC-H. Cells were plotted for their CD14 and CD16 expression and three distinct subpopulations of monocytes, i.e., classical, non-classical, and intermediate were analyzed. Resulting populations were subsequently plotted for CCR2 and CX3CR1 expressions. And finally, cells were selected for HLA expression. [file Image_1.jpeg]
